# Novel Coronavirus (COVID-19) Knowledge, Precaution Practice, and Associated Depression Symptoms among University Students in Korea, China, and Japan

**DOI:** 10.3390/ijerph17186671

**Published:** 2020-09-13

**Authors:** Bo Zhao, Fanlei Kong, Myo Nyein Aung, Motoyuki Yuasa, Eun Woo Nam

**Affiliations:** 1 Department of Health Administration, Graduate School, Yonsei University, 1 Yonseidae-gil, Wonju, Gangwon-do 26493, Korea; zhaobo@yonsei.ac.kr; 2 Centre for Health Management and Policy Research, School of Public Health, Cheeloo College of Medicine, Shandong University, Jinan 250012, China; 3 NHC Key Lab of Health Economics and Policy Research, Shandong University, Jinan 250012, China; 4 Juntendo Advanced Research Institute for Health Science, Juntendo University, Hongo 2-1-1, Bunkyo-ku, Tokyo 113-8421, Japan; myo@juntendo.ac.jp; 5 Global Health Service Course, Faculty of International Liberal Arts, Juntendo University, Hongo 2-1-1, Bumkyo-ku, Toyko 113-8421, Japan; moyuasa@juntendo.ac.jp; 6 Healthy City Research Center, Institute of Health and Welfare, Yonsei University, 1 Yonseidae-gil, Wonju, Gangwon-do 26493, Korea

**Keywords:** COVID-19, knowledge, Korea, China, Japan, university adults, preventative practices, depression

## Abstract

This study assessed university students’ knowledge and precaution practices of Novel Coronavirus (COVID-19) in South Korea, China, and Japan, and investigated their depressive states during the pandemic. This cross-sectional survey collected data from 821 respondents, using an anonymous online questionnaire designed by the Yonsei Global Health Center, from 23 March to 20 April 2020, which included socio-demographic questions, knowledge and perceptions of COVID-19, preventative practices, and the Patient Health Questionnaire-9 (PHQ-9) scale to assess mental health. High proportions of respondents showed good knowledge of the transmission pathways and information related to COVID-19. Contact history as well as concerns about family members and the disease showed statistically significant distinctions by nationality and gender. On the whole, all participants reported good levels of preventative practices. The Chinese group reported the highest preventative practice scores; and females scored higher than males. Moreover, the Japanese group showed the most severe depressive states; overall, females experienced more severe depression than males. Thus, authorities should especially emphasize the importance of COVID-19 precautions to males. Educational departments and health authorities should observe the mental health of university adults during the pandemic and plan interventions to improve it.

## 1. Introduction

Humans have been experiencing various kinds of health emergencies that have resulted from pandemic and epidemic diseases. Up to now, the World Health Organization (WHO) has published 101 issues of the Health Emergency, including health priorities and responses related to the current main health events [1]—the 2019 outbreak of the Coronavirus disease (COVID-19). It was first reported in December 2019 in Wuhan, China, and has since spread globally, resulting in an ongoing pandemic [2,3]. As of 21 May 2020, more than 5 million cases have been reported across 188 countries and territories, resulting in more than 332,000 deaths [4].

After the initial outbreak in China, the pandemic was first confirmed to have spread to Japan in January 2020 where cases have been recorded in each of the 47 prefectures except Iwate, resulting in over 10,000 cases by now (By 20 May 2020). The first confirmed case of the outbreak of COVID-19 in Korea was announced on 20 January 2020, and the number of confirmed cases rapidly increased [5]. At the end of February, China and Korea recorded the first and second-largest number of confirmed cases of COVID-19 in the world, respectively. On 20 March 2020, the Foreign Ministers of China, South Korea, and Japan held a video conference to discuss issues related to the Coronavirus and possible cooperation “to fight the deadly disease”. The three countries expressed their determination to join hands to overcome difficulties in the face of major challenges and their positive willingness to work together to fight the epidemic [6]. China and South Korea have since managed to control its domestic spread, but isolated cases still appear and are imported from overseas. Although Japan has somewhat flattened its infection curve, the number of cases has been slowly rising again [7,8]. Therefore, the number of COVID-19 cases continues to rise in Korea, China, and Japan (KCJ).

COVID-19 has relatively common symptoms with other types of Coronavirus, such as SARS and MERS, including fever, cough, fatigue, shortness of breath, and loss of smell and taste [9,10,11,12]. In addition, the virus can be primarily spread among people during close contact through the eyes, nose, and mouth, via droplets from coughing, sneezing, and talking with an infected person, and through contaminated surfaces [13,14]. Currently, there are no vaccines or specific antiviral treatments for COVID-19 available. Recommended measures to prevent the spread of the infection include frequent hand washing, maintaining physical distance from others, quarantine, covering one’s mouth while coughing, and keeping unwashed hands away from the face [15,16,17]. Some studies revealed that knowledge of the virus and the recommended precautions are the protective factors that may curb the infection rate [18,19,20]. Therefore, knowledge of transmission pathways of COVID-19 and other related information is needed so that the public can adopt the right precautionary measures before possible vaccines and drugs become available [21].

Given the relationships among the three countries from history, the three countries are influenced by the traditional Confucian culture and share certain similarities in the understanding of life and health [22]. Although the outbreak was first discovered in China, Korea and Japan are close to China and have a similar perception of the epidemic on physical space. Each KCJ country has adopted measures such as social distancing and wearing masks since the outbreak of COVID-19. In addition, China has relied on sealing its borders, mass testing, and enforcing regional lockdowns. Japan kept its borders open, had targeted testing, and a relaxed approach to social isolation, while South Korea also kept its borders open but imposed mass testing and loose social isolation. These measures have been protecting the population while mitigating the spread of COVID-19. However, studies showed that prolonged social isolation and loneliness are associated with potential public health problems, such as increased mortality risk and cognitive functional status decline [23,24,25,26]. It was also pointed out that a low-level of social activities can bring psychological stress and negative affect on the life quality of younger people [27,28]. With the continuous spread of the pandemic, delayed opening and online classes of colleges and universities in each KCJ country are expected to decrease the levels of social activities and then affect the mental health of college students. Therefore, as college students are considered to be the main force in the future of society, their mental status during the pandemic should also be approached seriously.

In spite of the cooperation of fighting against COVID-19 and responses are relatively easy to accept by the public, different strategies and measures that took by the three countries may lead to different levels of acquisition in COVID-19 information and preventative practices in individuals. However, no related comparison study among Korea, China and Japan about this disease has been conducted yet. Consequently, based on the importance of the role of the college adult group in society, it is important to ascertain their levels of knowledge and perceptions of COVID-19, as well as preventative practices and depression states among them. This comparative study of university adults in the three countries (KCJ) can provide fundamental information to improve the mental health of young adults and be helpful in understanding the differences in the knowledge and preventative practices related to COVID-19 among them.

## 2. Methods

### 2.1. Participants

**Figure 1:**
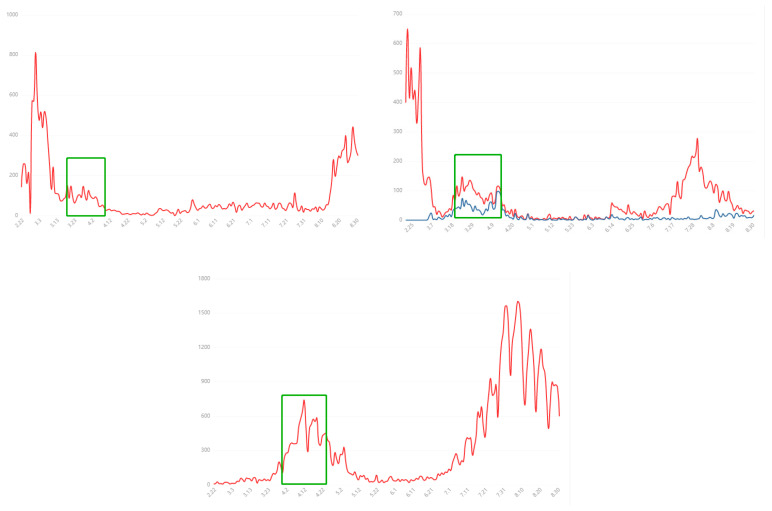
From left to right: Each day shows new cases reported since the previous day in Korea; Each day shows new cases reported since the previous day in China; Each day shows new cases reported since the previous day in Japan. (Note: Blue line: Input cases; Green Box: survey period; [29]).

The cross-sectional survey used an online questionnaire designed by Yonsei Global Health Center (YGHC) to collect data from respondents. The survey was conducted from 23 March to 20 April 2020 (Date as of 20 April 2020, resource from WHO; Korea: Total confirmed cases 10,674, Total deaths 236, Mortality Rate 0.022; China: Total confirmed cases 84,237, Total deaths 4642, Mortality Rate 0.055; Japan: Total confirmed cases 10,751, Total deaths 171, Mortality Rate 0.004. New cases changes refer to Figure 1) and involved a total of 821 university adults. Three-hundred-and-ninety individuals were from Korea (mean age: 23.14 ± 0.15, 41.54% male and 58.46% female), 281 from China (mean age: 23.63 ± 0.18, 29.54% male and 70.46% female), and 150 from Japan (mean age: 23.08 ± 4.78, 40% male and 60% female). The study was conducted collectively in the three countries.

### 2.2. Procedure

The COVID-19 questionnaire used in this study was first written in English after an exchange of opinions among the co-authors by focus group discussion. The questionnaire was discussed and designed by researchers to assess the knowledge, preventative behaviors, and mental health of university adults living in Korea, China, and Japan from a shared perspective.

Because of the limited resources and social distancing implemented following the COVID-19 outbreak, an online survey was given preference. With the help of collaborators and native speakers, Korean, Chinese, and Japanese versions of the questionnaire were created and hosted on survey platforms (Naver Form Tool in Korea, Surveystar in China, Google Form in Japan). In addition, a questionnaire pre-test was conducted among some students in each country before the actual survey to ensure that the questions being asked accurately reflect the information the researcher desires and that the respondent could and will answer the questions. Researchers in each country checked readability, comprehension, and convenience for the respondents to answer the on-line survey. Potential respondents were sent a specific link to participate in this questionnaire.

The survey was administered by a researcher of YGHC and coordinated by the professors in each country, and it was distributed via Uniform Resource Locator (URL) to researchers in each school to collect the data of purposive samples consisting of university students, together with a consent form including the research objective and security of anonymity. We ensured that consent forms had been completed and that the participants had been informed about the questionnaire before the survey was conducted.

It took 8–10 min for participants to answer all the questions, which was checked by the use of mobile devices’ responses. The URLs of the online questionnaires (Korean, Chinese, Japanese version) were firstly disseminated by the research members who shared the link with their labmates and partners through their social media (Korea: Kakao Talk, Email; China: Wechat, QQ; Japan: Line). In order to enlarge the sample size and involve more areas in each country, the cooperators and researchers in each country helped by inviting their colleagues and students from their universities, and lectures or professors in other universities to forward URLs to university students they know to take part in the survey. In the online survey questionnaire, we stipulated that the main questions in the survey (as shown below in Section 2.3) were mandatory questions, which means the participants have to complete all answers before they submitted the online questionnaire. This type of answer mode improved the quality of the questionnaire and the rate of answering questions. Therefore, the population data analyzed in this study were those respondents who answered all the questions and successfully handed over. However, students who failed to complete all the questions and were unable to submit the questionnaire were excluded from the data analysis. AS for the format of the question, there are single-selected questions, and multiple-selected questions, and filling in the blanks. In the end, we recruited 400 respondents from Korea, 281 from China, and 150 from Japan. During the data analysis, 10 respondents in the Korean group were found to have abnormal answers and then were deleted. Thus, the overall sample was 390 Korean, 281 Chinese, and 150 Japanese.

### 2.3. Measures

Several scales for measuring the knowledge, preventative practices, and mental states have been developed based on prior reviews [30]. Questions were found to own a reasonable validity and reliability in Wang’s research on the Chinese general population. Yonsei Global Health Center (YGHC) made some changes according to the specific situation in the three countries. The questionnaire related to the COVID-19 outbreak consisted of questions that covered several areas: (1) demographic and physical health data; (2) knowledge and perceptions of COVID-19; and (3) precautionary measures against COVID-19 in the past 14 days. The Patient Health Questionnaire-9 (PHQ-9) was added to the study.

- Demographic and physical health status data elicited from the respondents included gender, age, educational level, marital status, work status, self-assessed physical condition, chronic diseases, potential symptoms, travel abroad, and whether or not they were quarantined in the past 2 weeks.
- Knowledge of COVID-19 was assessed using items related to the transmission pathways (Agree, Disagree), the disease information (Yes, No), and the sources of COVID-19 information (Internet, TV, Radio, News, Family, Others). The questions on the perception of COVID-19 consisted of the history of contact (Yes, No/Not sure), information satisfaction (5 levels, ranging from “Dissatisfied” to “Satisfied”), diagnosis confidence (5 levels; “Not confident”—“Very confident”), probability of getting infected and surviving (5 levels; “Low—“High”), and concerns about family members and the disease (5 levels: “Low—“High”). High scores indicated a high level of certainty.
- Precautionary measures comprised of nine preventative practices. The norm was set by the degree of measures practiced on a daily basis (1 = “I never do this” to 5 = “I do this every day”), generating a total score of nine (9 × 1~9 × 5). Cronbach’s Alpha coefficient of preventive practices of the COVID-19 scale was 0.71.
- The PHQ-9, published by the American Psychiatric Association, was used to diagnose depressive symptoms based on the nine criteria for depression set by the Diagnostic and Statistical Manual of Mental Disorders [31]. Each item was rated on a 4-point scale ranging from 0 (“Not at all”) to 3 (“Almost every day”) [32]. The scoring criteria of PHQ-9 were divided into five groups: 0–4, 5–9, 10–14, 15–19, and 20–27, which corresponded to “minimal or none,” “mild,” “moderate,” “moderately severe,” and “severe” depression, respectively [33]. The higher the score, the more intense the level of depression. Cronbach’s Alpha coefficient of the scale in this study was 0.87.

### 2.4. Statistical Analysis

Descriptive analyses, χ^2^ tests, and ANOVA tests were conducted using SPSS (Statistical Package for the Social Sciences, IBM Corp. Released 2016. IBM SPSS Statistics for Windows, Version 24.0. IBM Corp., Armonk, NY, USA). Results were considered significant at a threshold of *p* < 0.05.

## 3. Results

### 3.1. General Demographic and Physical Health Data

Significant differences between the demographic data of the three nationalities were observed for “Age” (χ^2^ = 3.26, *p* < 0.05), “Educational level” (χ^2^ = 62.29, *p* < 0.001), “Marital status” (χ^2^ = 9.18, *p* < 0.05), “Job” (χ^2^ = 36.21, *p* < 0.001), “Traveled abroad” (χ^2^ = 8.69, *p* < 0.05), and “Self-quarantine” (χ^2^ = 166.64, *p* < 0.001). The proportion of graduates in China was 32.03% in total, which was higher than that in Korea (22.89%) and Japan (19.33%). However, the percentage of single participants in China (94.66%) was the lowest among the three (98.72% for Korea, and 96.00% for Japan). In terms of the respondents’ job status, Korea showed the highest proportion (94.87%) of participants who were current students, then followed by China (88.61%) and Japan (82%). Although there was a significant distinction among the three groups in terms of the number of participants who traveled abroad in the past 2 weeks, most of them (more than 95%) answered they did not go abroad. As for self-quarantine, 54.25% of the Chinese had experienced it in the 2 weeks preceding the study, which was significantly different from the Koreans (9.49%) and Japanese (24.00%) (Table 1).

In terms of “Symptoms of body discomfort in the past 14 days”, Japanese respondents showed the highest percentage (36.67%) of those who had one or more symptoms (such as persistent fever, chills, headaches, etc.), which was higher than that of the Koreans (32.54%) and Chinese (9.61%). Correspondingly, the self-assessed physical condition of the Chinese was in the top position among the three groups. In general, most of them (91.96%) reported a good level of physical condition without chronic diseases. 

### 3.2. Knowledge and Perception of COVID-19

Table 2 shows the comparison of knowledge and perception of COVID-19 among the respondents of the three study countries. Regarding the transmission routes of COVID-19, the fact that it can spread through droplets was known by 97.93% of the participants, followed by objects (83.68%) and air (49.09%). The levels of keeping up to date with relevant information in the three groups were similar, except for the knowledge of the Japanese individuals regarding the recovered cases. In particular, more Chinese respondents had a good knowledge of the transmission pathways and up to date information compared to Korean and Japanese respondents. Furthermore, almost 93.42% of the respondents got this information via one of the three following sources, the internet, TV, others. Moreover, the Japanese group had a relatively high proportion (20%) of those who had contact or suspected contact with the patients. It was also found that the Japanese had a comparatively low level of information satisfaction (42.67% was above satisfied) and diagnosis confidence (only 64.44% was highly confident). Besides, in terms of the perceived probability of getting infected, 81.11% of the Japanese respondents thought they may get infected, while it was only 33.08% and 19.93% for the Koreans and the Chinese respectively. The table also shows a distinction in the concerns regarding their family members between the three groups. Additionally, there were significant differences in the variables, except for the perceived probability of surviving after infection, among the nations. Differences were also found significantly related to “Recovered cases” (χ^2^ = 8.416, *p* < 0.01), “Contact history” (χ^2^ = 1.12, *p* < 0.05), “Concern about family members” (χ^2^ = 11.21, *p* < 0.01), and ‘Concern about disease” (χ^2^ = 10.63, *p* < 0.05) between males and females.

### 3.3. Preventative Practices against COVID-19

The average scores of the Chinese respondents for five out of the nine preventative practices were significantly higher than those of the Korean and Japanese respondents. Koreans reported a mean score of 4.47 in “Covering mouth when coughing and sneezing” while the Chinese and Japanese scored 4.38 and 4.21, respectively, and the distinction between the Korean group and Japanese group was statistically significant (*p* < 0.05). The practice of “Wearing masks regardless of the presence or absence of symptoms” differed significantly between all three groups (*p* < 0.001). The Japanese group showed higher scores than the other two groups only in “Washing hands with soap and water” (*F*(2) = 6.203, *p* < 0.01). Chinese individuals and Korean individuals got higher scores in “Washing hands immediately after coughing, rubbing nose or sneezing” (*p* < 0.001), and “Washing hands after touching contaminated objects” (*p* < 0.001) respectively. The Chinese group revealed better preventative actions in “Washing hands immediately after coughing, rubbing nose or sneezing” (*F*(2) = 8.10, *p* < 0.001), “Avoiding using elevators” (*F*(2) = 149.11, *p* < 0.001), “Sitting in one row while having a meal” (*F*(2) = 216.58, *p* < 0.001), and “Avoiding meeting with more than 10 people” (*F*(2) = 24.86, *p* < 0.001). Overall, the total mean scores of the Korean, Chinese, and Japanese respondents in preventative practices were 33.90 ± 5.28, 39.30 ± 5.46, and 34.39 ± 5.53, respectively. Thus, the Chinese group did relatively better than the other two groups (*p* < 0.001). Meanwhile, the performance comparisons between Males and Females also manifested statistically significant differences in five behaviors. Males’ precautions were not as good as those of Females’ (Table 3).

### 3.4. Respondents’ Depressive States

Based on the results of the PHQ-9 survey, the depression symptoms of the Japanese (7.33 ± 6.20, 95% CI: 6.33–8.33) were relatively worse than those of the Koreans (5.94 ± 5.44, 95% CI: 5.39–6.48) and Chinese (6.40 ± 5.12, 95% CI: 5.79–7.00), and the differences among the three countries were statistically significant (*F*(2) = 2.97, *p* < 0.05). The *t*-test between depression scores and gender revealed that the mean scores of males (5.83 ± 5.16) were lower than those of females (6.66 ± 5.67). Moreover, female respondents showed relatively severe depressive symptoms compared to male respondents (*t* = −2.09, *p* < 0.05). According to the classification criterion, the Korean group showed a big percentage of “Non-depressed” individuals (50.77%), while the Chinese group showed a high proportion of respondents in the “Mild” category (39.86%). The score levels of the Chinese and Japanese showed a similar pattern, that is males with “Mild” and “Moderate” depression levels accounted for a bigger percentage than the females, while females in the “Moderately severe” and “Severe” categories constituted higher proportions than males. In contrast, among the Korean participants, the females in “Mild”, “Moderate”, “Moderately severe”, and “Severe” categories all accounted for higher percentages than males. Moreover, the relationships between the different levels of the depressive symptoms showed by nationality (χ^2^ = 25.849, *p* < 0.001) and gender (χ^2^ = 10.630, *p* < 0.05) also demonstrated statistically significant differences (Table 4). 

## 4. Discussion

Since the first case was reported in 2019, COVID-19 has become one of the largest pandemics in the world involving more than 200 countries and regions and posing a significant threat to populations worldwide [34]. Given the importance of the knowledge of COVID-19 and effective preventative practices in reducing the infection rates and controlling the spread of the disease, and the current absence of treatment or a vaccine [35,36], this study was conducted to assess the knowledge of COVID-19 and related preventative actions of university adults, as well as to evaluate their depressive states amidst the outbreak in Korea, China, and Japan.

### 4.1. Knowledge and Preventative Practices

Although the respondents’ educational status and working conditions were significantly different among the three groups, it was reported that 92% did not have chronic diseases. The present findings indicated that around 80% of the respondents self-assessed as being in a good physical condition and without any uncomfortable symptoms in the past 2 weeks. Because the first COVID-19 outbreak occurred in China, we could see that more than half of the Chinese respondents had experienced self-quarantine, which means they had to stay at home or workplace due to the strict lockdown and restriction measures at that time [37]. Compared with the situations in Korea and Japan, the countries that have been taking different approaches to social distancing measures to tackle the COVID-19 pandemic, the lockdown deployed across China involved about 1.4 billion people and was a tough form of social distancing strategy [38].

The approach to the first wave of the COVID-19 outbreak in Japan was to request citizens’ cooperation in staying at home. An emergency state was declared for all 47 prefectures by the Government in April 2020, and financial support was deployed to individual households while essential social services kept running. Japanese citizens followed each local government’s guidelines and stayed at home during the emergency period from 7 April to 25 May 2020. The reasons for the people’s conformance with the guidelines were the high health literacy and their nature to obey regulations set by the government [39]. Meanwhile, the Korean government implemented the COVID-19 response system based on the 3 Ps (Preemptive, Prompt, and Precise) and 3 Ts (Test, Trace, and Treat) [40]. One of the key factors in the success of flattening the infection curve in Korea was continuous testing. Testing was scaled up aggressively (nearly 18,000 per day) in Korea. Apart from that, the authorities called for social distancing, isolation, as well as universal body temperature checks. Contact tracing was done through CCTV, credit card transactions, and car screening centers [41]. However, the mandatory quarantines and the intensive surveillance had effectively prevented further exportation of infected individuals to the rest of the country. Whether these different policies and approaches are applicable to different settings across the world has been questioned and observed by global public health experts and needs to be assessed further [42,43].

The study found that most respondents had a good knowledge of COVID-19. Study participants achieved high levels of awareness of the COVID-19 transmission pathways and updated information. Considering information satisfaction, 96% of Chinese individuals were “Above satisfied”, which was a much higher percentage than in Korea or Japan. The related health institutions and mess media do good publicity work. We interestingly found that many Japanese respondents hold the view that they had a fairly high risk of getting infected, and there were also higher proportions of being highly concerned about family members and being worried about “the disease” in the Japanese group than in the Korean and Chinese groups. Although Japan has a unitary government system, sub-national governments have gained a fair amount of autonomy in the policy-making process [44]. With the influence of the severe crisis of the Diamond Princess cruise ship [45], the relatively less strict response strategy attributed to its decentralized regime and tight cultural orientation [46] has controlled the epidemic as well as brought a degree of unease and panic to the public.

In addition, our findings highlighted the diversity of the preventative practices by nationality and gender. The significant differences between female and male respondents related to the precautions, which is also evidenced by the previous studies suggesting that the level of preventative measures of female adults tends to be higher than that of males [47,48,49]. In terms of nationality, the Chinese group showed the highest mean total score of precautions assessment. This can be explained by the fact that unprecedented measures have been adopted to control the rapid spread of COVID-19 in China, and the educational institutions and popular media contributed significantly to this [50,51]. However, the Japanese group reported better performance than the other two in “Washing hands with soap and water”. It is the prevention measure that WHO recommended to prevent COVID-19 transmission since the onset of the pandemic [52]. Hand hygiene has been a part of the basic education in Japan for many decades to develop this behavior as a healthy habit [53]. As a collective practice, this infection control behavior could be a contribution to the success in the containment of the first wave of the pandemic in Japan.

Therefore, as a result of the disparities in preventative practices between different countries and sex groups, each KCJ government should bring the importance of precautions to public attention via mass media and publicity departments, especially for the male group.

### 4.2. Depression Symptoms

Mental health issues increased significantly in young adults (18–25 years old) over the last decade. The rate of individuals reporting symptoms consistent with major depression in the last 12 months increased 63 percent in young adults age 18 to 25 from 2009 to 2017 (from 8.1 percent to 13.2 percent) in America [54]. Concerning the depression symptom found in the university adults in this study, the depressive status of females was relatively worse than that of males. The result is in conformity with the previous research, the aggregate prevalence was 14.4% (95% CI: 11.1% to 11.7%) for women and 11.5% (95% CI: 9% to 14.6%) for men [55]. This problem may be deeply rooted in female’s self-esteem and gender discrimination [56], like employment preference and body image [57,58]. Statistics show that Korea, China, and Japan are some of the most gender unequal countries in the world [59]. Women tend to be more influenced by the surrounding media and fashion industry than men [60], as well as suffering more selective preference in many fields. Especially Korea has maintained a unique style that has influenced worldwide trends known as the “K-Wave”, which also may push young Korean women to pursue fashion and, as a result, suffer from psychological distress. This may also explain why the proportion of Korean females in the four depression categories was larger than that of males, particularly with regard to the “Mild” and “Moderate” levels.

The mean score of the depressive status of the Japanese group was higher than that of the other two groups. This could be a consequence of losing their opportunity to host the 2020 Olympics in Tokyo. This finding converges with the previous study that suggested that the depression of adolescent school children in Japan was more severe than that in Korea and China [61]. In addition, studies also found that 98.7% of adolescents in metropolitan areas of Japan considered themselves as having mental health problems [62]. Furthermore, in each KCJ country, Confucianism has been shared and permeated for thousands of years [63]. Interpersonal harmony, relational hierarchy, and traditional conservatism are the values and principles that are generally emphasized in the three study countries [64]. Studies also demonstrated that Japanese females are more conservative than Japanese males [65], and, in general, the Japanese tend to avoid sharing their inner worries and feelings with others [61], which may also affect their mental state to a certain extent.

Depression symptoms may be affected by various determinants, especially, during the intense pandemic period. Scholars had presented some risk factors contributing to psychological problems, including a long time spent at home, poor health status, and high-level concern about COVID-19 [30]. As well, the importance of some protective factors (such as preventative measures) in relieving depression symptoms has also been previously highlighted [30,35,36]. Thus, for the students in this study, it is necessary for them to be aware of the depressive states. To prevent depressive problems from becoming more serious during the pandemic, educational institutions, health authorities, and the university departments of each KCJ government should offer mental health services and educational programs to the students and their families. Additionally, special attention should be paid to the female group during the pandemic. Digital health promotion and telemental health would be powerful tools for these purposes.

### 4.3. Limitations and Future Research

The empirical results reported here should be considered in light of some limitations. Firstly, the non-probability sampling method, which we applied in data collection may limit readers to generalize the findings into broader contexts, but this method is widely accepted in social and medical sciences when the target population is difficult to locate [66], especially in such a special pandemic situation. So it would be ideal to conduct a generalizable study with the aid of platforms and institutions among potential participants adopting probability methods like random sampling as well as to enlarge the sample size.

Secondly, only nationality and gender were assessed in the depression comparison of the study. Research targets could be tested further using samples from other groups in the three countries and more variables should be included such as socioeconomic and environmental factors. Future studies can also gather longitudinal data to examine the causality and interrelationships between variables that are important to participants’ mental health in the context of an outbreak of the virus.

Nevertheless, the strength of this study is that results from university students of KCJ countries about COVID-19 and its mental impact will be directly applicable to the reopening schools and resuming the new normal.

## 5. Conclusions

Based on the results of the study, we found that most respondents in the three student groups had a good knowledge of COVID-19. Although they showed different levels of concern about their family members and the disease across the three nationalities, they shared good performance of the preventative practices. Particularly, the Chinese group in this study showed better performance than the others, and so did female individuals when compared to males. The above results of this study demonstrate the need for the governments to emphasize the importance of precautions to public attention, especially for males. Along with, female students in this study reported a more severe degree of depression than male students in all three countries, with the Japanese participants showing the worst depressive states among them. It should be noted that mental health problems and non-communicable diseases among severe secondary outcomes are related to the COVID-19 pandemic.

To promote a mental health-friendly environment for these university students, some professional counseling and programs to address depression should be provided by university departments, local and central governments. Furthermore, more research should be conducted to ascertain the depression symptoms among other countries in the next few months during the COVID-19 pandemic to provide more generalizable data. The present results may offer fundamental information for the development of mental health programs for university adults as well as other groups.

## Figures and Tables

**Table 1:** General demographic and physical health characteristics (*n* = 821). Mean ± S.D. *n* (%).

Items	Korea			China			Japan			Total (M = 305, F = 516)	χ^2^
	Male (*N* = 162)	Female (*N* = 228)	Total (*N* = 390)	Male (*N* = 83)	Female (*N* = 198)	Total (*N* = 281)	Male (*N* = 60)	Female (*N* = 90)	Total (*N* = 150)		
Age	24.05 ± 0.25	22.50 ± 0.16	23.14 ± 0.15	23.57 ± 0.41	23.66 ± 0.19	23.63 ± 0.18	24.49 ± 0.59	23.86 ± 0.60	24.13 ± 0.53	23.08 ± 4.78	F = 3.248 *
Educational level											
Undergraduate	145 (89.51)	215 (94.30)	360 (92.31)	66 (77.11)	127 (64.14)	191 (97.97)	45 (75.00)	76 (84.44)	121 (80.67)	672 (81.85)	65.289 ***
Graduate	17 (10.49)	13 (5.70)	30 (7.69)	19 (22.89)	71 (35.86)	90 (32.03)	15 (25.00)	14 (15.56)	29 (19.33)	149 (19.15)	
Marital status											
Single	160 (98.77)	225 (98.68)	385 (98.72)	75 (90.36)	191 (96.46)	266 (94.66)	58 (96.67)	86 (95.56)	144 (96.00)	795 (96.83)	9.177 *
Married	2 (1.23)	3 (1.32)	5 (1.28)	8 (9.64)	7 (3.54)	15 (5.34)	2 (3.33)	4 (4.44)	6 (4.00)	26 (3.17)	
Job Status											
Students	155 (95.68)	215 (94.30)	370 (94.87)	76 (91.57)	173 (87.37)	249 (88.61)	53 (88.53)	70 (77.78)	123 (82.00)	742 (90.38)	36.212 ***
Working	4 (2.47)	11 (4.82)	15 (3.85)	6 (7.23)	20 (10.10)	26 (9.25)	5 (8.33)	16 (17.78)	21 (14.00)	62 (7.55)	
Other	3 (1.85)	2 (0.88)	5 (1.28)	1 (1.20)	5 (2.53)	6 (2.14)	2 (3.33)	4 (4.44)	6 (4.00)	17 (2.07)	
Traveled abroad											
No	159 (98.15)	228 (100)	387 (99.23)	80 (96.39)	195 (98.48)	285 (97.86)	57 (95.00)	86 (95.56)	143 (95.33)	805 (98.05)	8.689 *
Yes	3 (1.85)	0 (0)	3 (0.77)	3 (3.61)	3 (1.52)	6 (2.14)	3 (5.00)	4 (4.44)	7 (4.67)	16 (1.95)	
Self-quarantine											
No	149 (91.98)	204 (89.47)	353 (90.51)	34 (40.96)	94 (47.47)	128 (45.55)	44 (73.33)	70 (77.78)	114 (76.00)	595 (72.47)	166.640 ***
Yes	13 (8.02)	24 (10.53)	37 (9.49)	49 (59.04)	104 (52.53)	153 (54.45)	16 (16.67)	20 (22.22)	36 (24.00)	226 (17.53)	
Several symptoms											
No	118 (72.84)	149 (65.35)	267 (68.46)	73 (87.95)	181 (91.41)	254 (90.39)	39 (65)	56 (62.22)	95 (63.33)	616 (75.03)	55.329 ***
Yes	44 (27.16)	79 (34.65)	123 (32.54)	10 (12.05)	17 (8.59)	27 (9.61)	21 (35)	34 (37.78)	55 (36.67)	205 (24.97)	
Self-assessed physical condition											
Above good	117 (72.22)	166 (72.81)	283 (72.56)	73 (87.95)	184 92.93)	257 (91.46)	44 (73.33)	64 (71.11)	108 (72.00)	648 (78.93)	40.356 ***
Below fair	45 (27.78)	62 (27.19)	107 (27.44)	10 (12.05)	14 (7.07)	24 8.54)	16 (26.67)	16 (18.89)	42 (28.00)	173 31.07)	
Chronic diseases											
No	145 (89.51)	210 (92.11)	355 (91.03)	74 (89.16)	188 (94.95)	262 (93.24)	57 (95.00)	91 (90.00)	138 (92.00)	755 (91.96)	1.082
Yes	17 (10.49)	18 (7.89)	35 (8.97)	9 (10.84)	10 (5.05)	19 (6.76)	3 (5.00)	9 (10.00)	12 (8.00)	66 (8.04)	

* *p* < 0.05, *** *p* < 0.001.

**Table 2:** Respondents’ Knowledge and Perception of COVID-19, PHQ-9 level, and variable relationships by nationality and gender (*n* = 821) Mean ± S.D. *n* (%).

Items	Korea			China			Japan			Total (*N* = 821)	χ^2^ (by)	
	Male (*N* = 162)	Female (*N* = 228)	Total (*N* = 390)	Male (*N* = 83)	Female (*N* = 198)	Total (*N* = 281)	Male (*N* = 60)	Female (*N* = 90)	Total (*N* = 150)		Nationality	Gender
Transmission routes												
Droplets (agree)	153 (94.44)	224 (98.25)	377 (96.67)	83 (100)	197 (99.49)	280 (99.64)	59 (98.33)	88 (97.78)	147 (98.00)	804 (97.93)	7.145 *	3.493
Objects (agree)	121 (74.69)	178 (78.07)	299 (76.67)	76 (91.57)	184 (92.93)	260 (92.53)	52 (86.67)	76 (84.44)	128 (85.33)	687 (83.68)	30.448 ***	1.477
Air (agree)	71 (43.83)	93 (40.79)	164 (42.05)	59 (71.08)	128 (64.65)	187 (66.55)	17 (29.33)	35 (38.89)	52 (34.67)	403 (49.09)	54.487 ***	0.514
Updated information												
Infected case (yes)	157 (96.91)	222 (97.37)	379 (97.18)	83 (100)	198 (100)	271 (100)	58 (96.67)	89 (98.89)	147 (98.00)	807 (98.29)	12.161 **	1.007
Death case (yes)	153 (94.44)	219 (96.05)	372 (95.38)	81 (97.59)	198 (100)	279 (99.29)	58 (96.67)	87 (96.67)	145 (96.67)	796 (96.96)	8.482 *	2.425
Recovered case (yes)	135 (83.33)	210 (92.11)	345 (88.46)	81 (97.59)	196 (98.99)	277 (98.58)	40 (66.67)	62 (68.89)	102 (68.00)	724 (88.19)	87.810 ***	8.416 **
Number of information sources												
1~3	161 (99.38)	223 (97.81)	384 (98.46)	73 (87.95)	193 (92.42)	256 (91.10)	57 (95.00)	70 (77.78)	127 (84.67)	767 (93.42)	37.291 ***	3.119
4~6	1 (0.62)	5 (2.19)	6 (1.54)	10 (12.05)	15 (7.58)	25 (8.90)	3 (5.00)	20 (22.22)	23 (15.33)	54 (6.58)		
Contact history											40.132 ***	1.198 *
No	135 (83.33)	185 (81.14)	320 (82.05)	81 (97.59)	192 (96.57)	273 (97.15)	54 (90.00)	66 (73.33)	120 (80)	713 (86.84)		
Yes/Suspected	17 (16.66)	43 (18.86)	70 (17.95)	2 (2.41)	3 (2.92)	8 (2.85)	6 (10.00)	14 (26.67)	30 (20.00)	108 (13.16)		
Information satisfaction											150.837 ***	0.032
Above satisfied	115 (70.99)	151 (66.23)	266 (68.21)	79 (95.18)	191 (96.46)	270 (96.09)	30 (50.00)	34 (37.78)	64 (42.67)	600 (73.08)		
Below satisfied	47 (29.01)	77 (33.77)	124 (31.79)	4 (4.82)	7 (3.54)	11 (3.91)	30 (50.00)	56 (62.22)	86 (57.33)	221 (16.92)		
Confidence in diagnosis											31.733 ***	0.059
Highly confident	138 (85.19)	195 (85.53)	333 (85.38)	66 (79.52)	151 (76.26)	217 (77.22)	37 (61.67)	58 (64.44)	95 (63.33)	645 (78.56)		
Lowly confident	24 (14.81)	33 (14.47)	57 (14.62)	17 (20.48)	47 (23.74)	64 (22.78)	23 (38.33)	32 (35.56)	55 (36.67)	176 (21.44)		
Perceived probability												
Getting infected (high)	48 (29.63)	81 (35.53)	129 (33.08)	25 (30.12)	31 (15.66)	56 (19.93)	43 (71.67)	73 (81.11)	116 (77.33)	301 (36.66)	142.894 ***	0.392
Surviving after infection (high)	130 (80.25)	190 (83.33)	320 (82.05)	72 (86.75)	173 (87.37)	245 (87.19)	55 (91.67)	74 (82.22)	129 (86.00)	694 (84.53)	3.599	0.027
Concerns about family members											18.261 ***	11.213 **
High	29 (17.90)	26 (11.40)	335 (85.90)	62 (74.70)	156 (78.79)	218 (77.58)	58 (96.67)	81 (90.00)	139 (92.67)	692 (84.29)		
Low	133 (82.10)	202 (88.60)	55 (14.10)	21 (25.30)	42 (21.21)	63 (22.42)	2 (3.33)	9 (10.00)	11 (7.33)	129 (15.71)		
Concerns about the disease											6.404 *	10.630 *
High	128 (79.01)	206 (90.35)	334 (85.64)	70 (84.37)	180 (90.91)	250 (88.97)	56 (93.33)	84 (93.33)	140 (93.33)	724 (88.19)		
Low	34 (20.99)	22 (9.65)	56 (14.36)	13 (15.66)	18 (9.09)	31 (11.03)	4 (6.67)	6 (6.67)	10 (6.67)	97 (11.81)		

* *p* < 0.05, ** *p* < 0.01, *** *p* < 0.001.

**Table 3:** Preventative practices of respondents and variable relationships by nationality and gender (*n* = 821). Mean ± S.D.

Items	Korea (*N* = 390)			China (*N* = 281)			Japan (*N* = 150)			Main Effect (by)				
										Nation				Gender
	Mean ± S.D.	95% Conf. Interval		Mean ± S.D.	95% Conf. Interval		Mean ± S.D.	95% Conf. Interval		F ^a^	Korea × China ^b^	Korea × Japan ^b^	China × Japan ^b^	*t*(M) ^c^
1: Covering mouth when coughing and sneezing	4.47 ± 0.91	4.38	4.56	4.38 ± 1.02	4.26	4.50	4.21 ± 1.18	4.02	4.40	3.031 *	0.088 ± 0.076	0.256 ± 0.107 *	0.167 ± 0.114	−1.286
2: Wearing a mask regardless of the presence or absence of symptoms	3.18 ± 1.48	3.04	3.33	4.25 ± 0.91	4.15	4.36	3.57 ± 1.50	3.33	3.82	69.781 ***	−1.071 ± 0.092 ***	−0.391 ± 0.144 *	0.679 ± 0.134 ***	−2.125 *
3: Washing hands with soap and water	4.76 ± 0.59	4.70	4.82	4.60 ± 0.68	4.52	4.69	4.81 ± 0.63	4.71	4.91	6.203 **	0.151 ± 0.051 **	−0.050 ± 0.058	−0.202 ± 0.064 **	−4.710 ***
4: Washing hands immediately after coughing, rubbing nose or sneezing	3.65 ± 1.20	3.53	3.77	3.99 ± 1.14	3.85	4.12	3.61 ± 1.375	3.38	3.83	8.099 ***	−0.337 ± 0.091 ***	0.042 ± 0.128	0.379 ± 0.131 *	−1.342
5: Washing hands after touching contaminated objects	4.71 ± 0.71	4.64	4.78	4.47 ± 0.80	4.38	4.56	4.27 ± 1.20	4.08	4.47	13.933 ***	0.241 ± 0.060 ***	0.437 ± 0.105 ***	0.196 ± 0.109	−2.315 *
6: Avoiding public transportation	4.43 ± 0.95	4.33	4.52	4.57 ± 0.74	4.49	4.66	4.43 ± 0.97	4.28	4.59	2.474	−0.147 ± 0.065	−0.008 ± 0.092	0.140 ± 0.090	−3.167 **
7: Avoiding using elevators	2.34 ± 1.51	2.19	2.49	4.12 ± 1.24	3.97	4.26	2.64 ± 1.66	2.37	2.91	149.119 ***	−1.779 ± 0.106 ***	−0.302 ± 0.156	1.477 ± 0.155 ***	−2.912 **
8: Sitting in one row while having a meal	2.14 ± 1.43	1.99	2.28	4.19 ± 1.19	4.05	4.32	2.47 ± 1.63	2.21	2.74	216.586 ***	−2.049 ± 0.101 ***	−0.337 ± 0.151	1.712 ± 0.150 ***	−0.524
9: Avoiding meeting with more than 10 people	4.23 ± 1.23	4.11	4.35	4.73 ± 0.68	4.65	4.81	4.37 ± 1.21	4.17	4.56	24.864 ***	−0.502 ± 0.074 ***	−0.136 ± 0.117	0.366 ± 0.107 **	−1.98 *
Questions Sum Scores	33.90 ± 5.28	33.37	34.42	39.30 ± 5.46	38.66	39.94	34.39 ± 5.53	33.49	35.28	88.850 ***	−5.405 ± 0.421 ***	−0.489 ± 0.525	4.916 ± 0.557 ***	−3.793 ***

Note: a: F test; b: post-hoc test; c: *t*-test. * *p* < 0.05, ** *p* < 0.01, *** *p* < 0.001.

**Table 4:** Relationships between the level of depressive symptoms by nationality and gender.

Items	Korea (*N* = 390)		China (*N* = 281)		Japan (*N* = 150)		Total	Main Effect (by)	
								Nationality	Gender (M)
Sum scores	5.94 ± 5.44 (CI: 5.39–6.48)		6.40 ± 5.12 (CI: 5.79–7.00)		7.33 ± 6.20 (CI: 6.33–8.33)		6.35 ± 5.50	F ^a^ = 2.970 *	T ^b^ = −2.091 *
0–4 (Non-depressed)	M: 96 (59.26) F:102 (44.74)	198 (50.77)	M: 23 (27.71) F: 88 (44.44)	111 (39.50)	M: 24 (40.00) F: 36 (40.00)	60 (40.00)	369 (44.95)	χ^2^ = 25.849 ***	χ^2^ = 10.630 *
5–9 (Mild)	M: 41 (25.31) F: 64 (28.07)	105 (26.92)	M: 39 (46.99) F: 73 (36.87)	112 (39.86)	M: 18 (30.00) F: 23 (25.56)	41 (27.33)	258 (31.43)		
10–14 (Moderate)	M: 19 (11.73) F: 34 (14.91)	53 (13.59)	M: 16 (19.28) F: 23 (11.62)	39 (13.88)	M: 13 (21.67) F: 12 (13.33)	25 (16.67)	117 (14.25)		
15–19 (Moderately severe)	M: 3 (1.85) F: 22 (9.65)	25 (6.41)	M: 4 (4.82) F: 10 (5.05)	14 (4.98)	M: 3 (5.00) F: 13 (14.44)	16 (10.67)	55 6.69)		
20–27 (Severe)	M: 3 (1.85) F: 6 (2.63)	9 (2.31)	M: 1 (1.20) F: 4 (2.02)	5 (1.78)	M: 3 (3.33) F: 6 (6.67)	8 (5.33)	22 2.68)		

Note: a: Welch test; b: *t*-test; χ^2^: Chi-square test. Mean ± S.D. *n* (%), * *p* < 0.05, *** *p* < 0.001.
